# Nutritional quality of plant-based beverages in Türkiye: application of the updated nutri-score algorithm

**DOI:** 10.3389/fnut.2026.1832771

**Published:** 2026-05-18

**Authors:** Merve Ekici, Murat Bas

**Affiliations:** 1 Department of Nutrition and Dietetics, Faculty of Health Sciences, Agri Ibrahim Cecen University, Agri, Türkiye; 2 Department of Nutrition and Dietetics, Graduate School of Health Sciences, Acibadem Mehmet Ali Aydınlar University, Istanbul, Türkiye; 3 Department of Nutrition and Dietetics, Faculty of Health Sciences, Acibadem Mehmet Ali Aydınlar University, Istanbul, Türkiye

**Keywords:** front-of-pack labeling (FOPL), nutri-score, nutritional quality, plant-based beverages, product classification

## Abstract

**Background:**

This study aimed to evaluate the nutritional quality of plant-based beverages identified in selected national and local retail outlets in Türkiye using the updated Nutri-Score beverage algorithm.

**Methods:**

This cross-sectional descriptive study analyzed 56 plant-based beverage products from 10 brands identified between October and November 2025. Nutritional composition and Nutri-Score classifications were summarized by plant source and compared across product subtypes.

**Results:**

Oat-based beverages had the highest median energy and sugars, coconut-based beverages had the highest median saturated fat content, and soy-based beverages had the highest median protein content by plant source. Across product subtypes, statistically significant differences were observed in energy (*P* = 0.0004), sugars (*P* < 0.001), and the percentage of fruits, vegetables and legumes (*P* = 0.028). Barista products had the highest median energy value, whereas unsweetened products had the lowest median energy and sugars. Nutri-Score class distribution differed significantly across product subtypes (*P* < 0.001). Most unsweetened products were classified as Nutri-Score B (87.5%), whereas plain/original (71.87%) and barista-type (87.5%) products were predominantly classified as Nutri-Score C.

**Conclusion:**

Among the sampled plant-based beverages, product formulation appeared to be more strongly reflected in Nutri-Score classification than plant source.

## 1 Introduction

Plant-based beverages are defined as beverages derived from legumes, oilseeds, cereals, or pseudocereals that resemble cow's milk in terms of physical appearance, intended use, and sensory properties (1). In recent years, the consumption of these products has increased markedly on a global scale. This growth has been primarily driven by the rising prevalence of lactose intolerance and cow's milk protein allergy, the widespread adoption of vegan and vegetarian dietary patterns, increasing concerns regarding environmental sustainability, and ethical considerations related to animal-derived food production (2, 3). In particular, the reduction of greenhouse gas emissions, the limitation of the environmental burden associated with animal agriculture, and the promotion of sustainable dietary models have been identified as key factors contributing to the growing interest in plant-based beverages (1).

From a nutritional perspective, substantial differences exist between plant-based beverages and cow's milk. Cow's milk is widely recognized as a rich source of high biological value protein, calcium, phosphorus, and B-group vitamins (4). In contrast, the nutritional composition of plant-based beverages varies considerably depending on the botanical source used and the processing technologies applied during production. For instance, almond- and rice-based beverages typically contain low amounts of protein, whereas soy-based products can achieve protein levels that are closer to those of cow's milk (5). Moreover, most plant-based beverages do not naturally contain calcium or vitamin B1_2_; therefore, these micronutrients are commonly added through fortification (4). However, variability in fortification practices leads to substantial differences in nutritional profiles among products and may hinder consumers' ability to accurately evaluate their true nutritional value (5).

In addition, many plant-based beverages contain added sugars, salt, and stabilizing agents, which has raised concerns regarding their overall nutritional quality (6). This situation challenges the widespread perception that plant-based beverages are inherently “healthier” options in all circumstances. Nevertheless, these products are often perceived by consumers as healthier alternatives because they are cholesterol-free, generally lower in saturated fat, may contain bioactive and antioxidant compounds depending on the plant source, and are considered to be more easily digestible (3). Such perceptions are particularly influential among younger consumer groups (7) and have contributed significantly to the rapid increase in plant-based beverage consumption.

The growing popularity of plant-based beverages highlights the need for objective and comparable methods to assess their nutritional quality. In this context, front-of-pack nutrition labeling (FOPL) systems have gained increasing importance as tools that enable a holistic evaluation of the nutritional profile of foods. The Nutri-Score system, which is widely used in Europe, classifies foods from A (highest nutritional quality) to E (lowest nutritional quality) based on their contents of energy, sugars, saturated fat, and salt, as well as positive components such as protein, dietary fiber, and the proportion of fruits and vegetables (8). In recent years, the Nutri-Score algorithm has been updated, particularly for beverages and plant-based beverages, with the aim of more accurately reflecting their nutritional profiles (9).

The primary objective of the Nutri-Score system is to facilitate healthier food choices by consumers and to encourage the food industry to reformulate products toward more balanced nutritional compositions. Previous studies have demonstrated that Nutri-Score labeling contributes to consumers' preference for products with lower energy and sugar contents (8, 10, 11). However, the evaluation of plant-based beverages remains relatively complex. These products are classified within the beverage category, and their Nutri-Score ratings can vary substantially depending on sugar content, protein levels, and fortification status (9, 12). Despite the rapid growth of plant-based beverage consumption in Türkiye, there is a lack of comprehensive studies evaluating the nutritional profiles of these products using the updated Nutri-Score system. Therefore, the aim of the present study was to assess the nutritional quality of plant-based beverages identified in selected national and local retail outlets in Türkiye by applying the Nutri-Score algorithm. The findings are expected to contribute to more informed consumer choices and to support the development of evidence-based food labeling policies in Türkiye.

## 2 Materials and methods

### 2.1 Study design and sample selection

This cross-sectional descriptive study evaluated the nutritional profiles of plant-based beverages identified in selected national and local retail outlets in Türkiye using the updated Nutri-Score beverage algorithm.

Data collection was conducted between October and November 2025. The study sample consisted of a total of 56 plant-based beverages belonging to 10 different brands that were widely sold in Türkiye.

Products were included according to the following criteria: (i) being a plant-based beverage, (ii) being actively available for sale during the data collection period, and (iii) having a mandatory nutrition declaration and ingredient list.

The study sample was divided into groups according to plant-based source and product subtype. The study sample was grouped into eight plant-source categories: almond (*n* = 16), oat (*n* = 11), soy (*n* = 4), coconut (*n* = 9), hazelnut (*n* = 10), cashew (*n* = 2), pistachio (*n* = 2), and mixed (*n* = 2). The products were grouped into three market-oriented subtypes based on label wording and product positioning: unsweetened, barista, and plain/original. “Unsweetened” referred to products explicitly labeled as unsweetened or no added sugar. “Barista” referred to products specifically marketed for coffee use, barista applications, or foaming performance. “Plain/original” referred to standard drinking products that were neither labeled as unsweetened nor marketed as barista formulations.

Nutritional information was collected from product labels obtained through online supermarket databases and in-person store visits conducted in Türkiye's well-established national and premium supermarket chains, which represent a major share of the packaged food retail market. These outlets were selected because they represented the major retail channels in which plant-based beverages were commonly available during the study period. During data collection, we aimed to capture the full spectrum of this group by surveying both online and physical retail channels. Products derived from different plant sources (e.g., soy, almond, oat, rice, coconut) within the same brand were considered as separate items. Repeated observations of the same product across different stores were counted only once, and different package sizes of the same formulation were excluded. For products sold in concentrated form, the nutritional values were calculated based on the preparation instructions provided on the package and expressed per 100 ml of the ready-to-consume beverage. For each product, the following information was recorded: plant source, energy, saturated fat, sugars, protein, salt, presence of sweeteners, dietary fiber, and the percentage of fruits, vegetables and legumes. All nutritional values were standardized to 100 ml to ensure consistency with the Nutri-Score calculation requirements.

### 2.2 Nutri-score calculation

The nutritional quality of the products was assessed using the updated Nutri-Score nutrient profiling algorithm specifically developed for beverages and widely implemented across the European Union (9).

In accordance with the algorithm, negative points were assigned for energy, sugars, saturated fat, salt, and sweetener content, while positive points were assigned for dietary fiber, protein, and the percentage of fruits, vegetables and legumes. Dietary fiber is not mandatory on all product labels. Therefore, when fiber information was unavailable on the package and could not be verified from the manufacturer's official website, fiber was recorded as 0 g/100 ml for label-based Nutri-Score calculation. No products contained non-nutritive sweeteners according to the label information. Salt values were recorded directly when declared on the label. When only sodium was reported, salt was calculated as sodium × 2.5.

The overall Nutri-Score was calculated by subtracting the total positive points from the total negative points. Based on the final score, products were classified into five Nutri-Score categories: A (water), B (≤ 2), C (3 to 6), D (7 to 9), E (≥ 10). Accordingly, class A represents the highest nutritional quality, class B indicates good nutritional quality, class C moderate nutritional quality, class D low nutritional quality, and class E very low nutritional quality. Products were evaluated and interpreted based on this classification framework.

### 2.3 Ethical considerations

Ethical approval was not required for this study because all data were obtained from publicly available product labels and manufacturer-provided information, and no human or animal subjects were involved.

### 2.4 Statistical Analysis

All data were organized using Microsoft Excel and subsequently analyzed with SPSS software (version 25.0; IBM Corp., Armonk, NY, USA) (13). Normality of continuous variables was assessed using the Shapiro–Wilk test. As most continuous variables were not normally distributed, descriptive statistics are presented as median (25th−75th percentile), while categorical variables are expressed as frequencies (*n*) and percentages (%). Nutritional composition and Nutri-Score distributions were summarized according to plant source and product subtype. Because several plant-source subgroups included only a small number of products, comparisons across plant-source categories were treated as descriptive and exploratory. For product subtype comparisons, continuous variables were analyzed using the Kruskal–Wallis test. Categorical associations, including Nutri-Score class distributions across product subtypes, were evaluated using the Fisher–Freeman–Halton exact test where appropriate. A *P*-value of less than 0.05 was considered statistically significant.

## 3 Results

A total of 56 plant-based beverages were included in the study. The distribution of products according to plant source and product subtype is presented in Table 1. Of all products, 16 products (28.6%) were classified as unsweetened, 32 (57.1%) as plain/original, and 8 (14.3%) as barista-type. Almond-based beverages represented the largest plant-source category (*n* = 16), followed by oat (*n* = 11), hazelnut (*n* = 10), coconut (*n* = 9), and soy (*n* = 4), whereas cashew, pistachio, and mixed products were represented by two items each. Overall, almond- and oat-based beverages were the most frequently identified product categories in the sampled dataset. It is important to note that the relatively small number of soy (*n* = 4), cashew (*n* = 2), pistachio (*n* = 2), and mixed products (*n* = 2) in our sample may reflect their more limited availability within the sampled retail channels.

**Table 1:** Distribution of plant-based beverages included in the study according to product subtype and plant source.

Category	Unsweetened (*n*)	Plain/original (*n*)	Barista (*n*)	Total (*n*)
Almond	5	9	2	16
Oat	1	6	4	11
Soy	1	2	1	4
Coconut	3	5	1	9
Hazelnut	3	7	0	10
Cashew	2	0	0	2
Pistachio	1	1	0	2
Mixed	0	2	0	2
**Total**	16	32	8	**56**

Bold values indicate statistically significant *p*-values (*p* < 0.05).

The nutritional composition of products by plant source is presented descriptively in Table 2. Oat-based beverages showed the highest median energy (192.0 kJ/100 ml) and sugars (3.30 g/100 ml), whereas coconut-based beverages had the highest median saturated fat content (1.10 g/100 ml).

**Table 2:** Nutritional composition and Nutri-Score distribution per 100 ml of plant-based beverages by plant source.

Variable	Almond (*n* = 16)	Oat (*n* = 11)	Soy (*n* = 4)	Coconut (*n* = 9)	Hazelnut (*n* = 10)	Cashew (*n* = 2)	Pistachio (*n* = 2)	Mixed (*n* = 2)
Energy (kJ)	100.0 (96.0–125.2)	192.0 (185.5–219.0)	125.0 (117.8–127.2)	93.0 (80.0–123.0)	124.0 (110.0–142.5)	92.0 (92.0–92.0)	126.0 (117.0–135.0)	98.5 (98.2–98.8)
Sugar (g)	2.40 (0.20–3.20)	3.30 (2.15–3.55)	2.55 (1.80–2.73)	2.20 (0.20–2.70)	3.00 (0.68–3.35)	0.22 (0.21–0.23)	1.70 (0.95–2.45)	2.10 (1.90–2.30)
Saturated fat (g)	0.10 (0.10–0.20)	0.20 (0.15–0.30)	0.20 (0.18–0.30)	1.10 (0.90–1.50)	0.20 (0.11–0.20)	0.31 (0.30–0.32)	0.20 (0.20–0.20)	0.35 (0.23–0.47)
Salt (g)	0.10 (0.10–0.14)	0.10 (0.10–0.10)	0.10 (0.08–0.10)	0.10 (0.07–0.11)	0.10 (0.10–0.18)	0.62 (0.31–0.94)	0.10 (0.10–0.10)	0.11 (0.11–0.12)
Protein (g)	0.60 (0.50–0.80)	0.80 (0.60–1.00)	1.95 (1.72–2.00)	0.10 (0.10–0.30)	0.60 (0.43–0.60)	0.76 (0.74–0.78)	0.85 (0.83–0.88)	0.35 (0.33–0.38)
Fiber (g)	0.00 (0.00–0.33)	0.60 (0.00–1.36)	0.00 (0.00–0.00)	0.00 (0.00–0.00)	0.00 (0.00–0.22)	0.26 (0.19–0.33)	0.00 (0.00–0.00)	0.75 (0.38–1.12)
Fruits, vegetables and legumes (%)	2.35 (2.30–2.92)	9.90 (9.55–10.00)	2.80 (2.72–3.35)	5.30 (5.00–7.00)	2.65 (2.12–3.29)	0.00 (0.00–0.00)	3.75 (3.62–3.88)	4.80 (4.75–4.85)
Nutri-Score B *n* (%)	7 (43.75)	1 (9.09)	3 (75.0)	3 (33.33)	3 (30.0)	2 (100.0)	1 (50.0)	0
Nutri-Score C *n* (%)	7 (43.75)	9 (81.82)	1 (25.0)	5 (55.56)	7 (70.0)	0	1 (50.0)	2 (100)
Nutri-Score D *n* (%)	2 (12.5)	1 (9.09)	0	1 (11.11)	0	0	0	0

Values are presented as median (25th−75th percentile) for continuous variables and n (%) for Nutri-Score classes. Comparisons across plant-source categories are presented descriptively and should be interpreted as exploratory because several subgroups contained a limited number of products. Bold values indicate statistically significant *p*-values (*p* < 0.05).

Soy-based beverages had the highest median protein content (1.95 g/100 ml), while coconut-based beverages showed the lowest median protein value (0.10 g/100 ml). With respect to fiber, the highest median value was observed in mixed products (0.75 g/100 ml), followed by oat-based beverages (0.60 g/100 ml). The proportion of fruits, vegetables and legumes was highest in oat-based beverages (9.90 %), whereas cashew-based products had a median value of 0.00 %. These plant-source patterns should be interpreted cautiously because several subgroups contained a limited number of products.

Nutri-Score distributions also varied across plant sources. Among almond-based beverages, 43.75% were classified as Nutri-Score B, 43.75% as C, and 12.5% as D. Oat-based beverages were predominantly classified as C (81.82%), whereas most soy-based beverages were classified as B (75.0%). All cashew-based beverages were classified as B, while all mixed products were classified as C. Coconut- and hazelnut-based beverages were also predominantly classified as C, accounting for 55.56% and 70.0% of products in these groups, respectively.

When beverages were compared according to product subtype (Table 3), statistically significant differences were observed in energy content (*P* = 0.0004), sugars (*P* < 0.001), and the percentage of fruits, vegetables and legumes (*P* = 0.028). Barista products had the highest median energy value (179.5 kJ/100 ml), whereas unsweetened products had the lowest (98.0 kJ/100 ml). Median sugars were lowest in unsweetened products (0.20 g/100 ml) and higher in both plain/original (3.10 g/100 ml) and barista products (3.00 g/100 ml). The median percentage of fruits, vegetables and legumes was highest in barista products (8.35 %), followed by plain/original (4.10 %) and unsweetened products (2.65 %). No statistically significant differences were detected across subtype groups for saturated fat, salt, protein, or fiber (*P* > 0.05).

**Table 3:** Comparison of nutritional composition and Nutri-Score classifications per 100 ml by product subtype.

Variable	Unsweetened (*n* = 16)	Plain/Original (*n* = 32)	Barista (*n* = 8)	*P*
Energy (kJ)	98.0 (89.0–105.0)	127.5 (102.0–170.8)	179.5 (123.8–222.0)	**0.0004** ^*^
Sugar (g)	0.20 (0.15–0.20)	3.10 (2.40–3.48)	3.00 (2.58–3.48)	**<0.001** ^*^
Saturated fat (g)	0.20 (0.15–0.30)	0.20 (0.10–0.32)	0.30 (0.21–0.38)	0.458^*^
Salt (g)	0.10 (0.09–0.11)	0.10 (0.10–0.13)	0.10 (0.10–0.10)	0.574^*^
Protein (g)	0.65 (0.55–0.80)	0.50 (0.38–0.72)	0.90 (0.50–1.05)	0.131^*^
Fiber (g)	0.06 (0.00–0.40)	0.00 (0.00–0.30)	0.00 (0.00–0.48)	0.590^*^
Fruits, vegetables and legumes (%)	2.65 (0.00–4.00)	4.10 (2.50–5.48)	8.35 (2.72–10.00)	**0.028** ^*^
Nutri-Score B *n* (%)	14 (87.5)	6 (18.75)	0	<0.001^**^
Nutri-Score C *n* (%)	2 (12.5)	23 (71.87)	7 (87.5)	
Nutri-Score D *n* (%)	0 (0)	3 (9.37)	1 (12.5)	

Values are presented as median (25th−75th percentile) for continuous variables and n (%) for Nutri-Score classes. ^*^ Kruskal–Wallis test, ^**^ Fisher–Freeman–Halton exact test. Bold values indicate statistically significant *p*-values (*p* < 0.05).

Nutri-Score class distribution differed significantly across product subtypes (Fisher–Freeman–Halton exact test, *P* < 0.001). Most unsweetened beverages were classified as Nutri-Score B (87.5%), and none were classified as D. In contrast, plain/original beverages were predominantly classified as C (71.87%), with smaller proportions classified as B (18.75%) or D (9.37%). Barista products were also mainly classified as C (87.5%), whereas the remaining 12.5% were classified as D; none of the barista products received a B classification. Taken together, these findings indicate that subtype-related variation, particularly in energy and sugars, was reflected in Nutri-Score classification patterns among the sampled products.

## 4 Discussion

This study evaluated a sample of 56 plant-based beverages from 10 brands identified in selected retail outlets in Türkiye using the updated Nutri-Score beverage algorithm. The sampled products showed considerable variability in nutritional composition. While plant-source patterns were examined descriptively, subtype-related differences were more clearly reflected in the comparative analyses. The findings related to less represented plant-source categories should be interpreted cautiously due to the small number of products in these subgroups.

Descriptive examination of the sampled products indicated that oat-based beverages had the highest median energy value, while sugar values were also relatively high in this group. This pattern may be related to the technological characteristics of oat beverage production. The manufacturing of oat-based beverages commonly involves enzymatic processes, such as liquefaction and saccharification, which convert starch into more soluble carbohydrate fractions in order to improve suspension stability and sensory properties (14, 15). As a result, oat-based beverages may acquire a naturally sweeter taste and a higher energy density (16). In the 2023 update of the Nutri-Score algorithm for beverages, the scoring scale for the sugar component was restructured to allow a more sensitive differentiation between products with low sugar contents (9). The fact that 81.82% of oat-based products in the present study were classified as Nutri-Score C is therefore consistent with the contribution of both energy and sugars to less favorable scores.

The higher median energy content observed in barista-type products compared with unsweetened products highlights the importance of formulation-related differences. Added vegetable oils, stabilizing ingredients, sweetness profile, and processing design for specific applications such as coffee use may substantially alter the energy, sugar, fat, or fiber content of products within the same broad plant-source category. Barista formulations are specifically designed to ensure stability in coffee and to facilitate micro-foam formation; therefore, they often include additional ingredients that may increase overall energy density (17). The predominance of Nutri-Score categories C (87.5%) and D (12.5%) among barista products in the present sample is consistent with this pattern.

Statistically significant differences in sugars were observed across product subtypes. While unsweetened products contained negligible amounts of sugar, plain/original and barista products contained substantially higher levels. These findings suggest that, despite their generally healthy image, some plant-based beverages may contribute appreciably to total sugar intake depending on product formulation. In the 2023 beverage update of the Nutri-Score algorithm, the scoring scale for sugar was redefined in order to enhance discrimination between products with different sugar contents (9). Accordingly, the finding that most plain/original products were classified in category C (71.87%), whereas most unsweetened products were classified in category B (87.5%), can be explained by their lower energy and sugar contents.

A notable descriptive pattern in the sampled products was that coconut-based products showed the highest median saturated fat content among the sampled plant-source groups. The saturated fat in coconut is predominantly composed of lauric acid, whose effects on blood lipid profiles have been widely debated. Nevertheless, recent meta-analyses have demonstrated that coconut oil consumption increases LDL cholesterol concentrations, particularly when compared with unsaturated vegetable oils (18). Since saturated fat is negatively weighted in the Nutri-Score algorithm, it is not surprising that 55.56% of coconut-based products were classified in category C and 11.11% in category D.

Another notable pattern in the sampled products was the higher median protein content of soy-based beverages compared with most other plant-source groups. This observation is consistent with previous literature (19, 20). In the Nutri-Score algorithm, protein contributes positively to the final score (9) and the finding that 75% of soy-based beverages were classified as category B is compatible with this more favorable nutrient profile.

It is important to note that Nutri-Score incorporates protein as a favorable component based on quantity, but it does not evaluate protein quality, amino acid profile, or digestibility (8, 9). This distinction is particularly relevant for plant-based beverages, because products with similar protein values may differ substantially in their nutritional contribution depending on botanical source and protein quality. Therefore, a more favorable Nutri-Score driven partly by protein content should not be interpreted as a complete indicator of protein adequacy.

Studies conducted in the United States (21) and Canada (22) have similarly reported substantial variability in the nutritional composition of plant-based beverages. The U.S. study found that plant-based beverages available in the market vary considerably in terms of protein, sugar, and enrichment practices (21). Likewise, many plant-based beverages in the Canadian food supply contain lower protein and, in some cases, higher sugar than their conventional counterparts, with substantial differences observed across product categories (22). These findings are broadly consistent with the present sample, in which unsweetened products tended to receive more favorable Nutri-Score classifications, whereas barista formulations tended to receive less favorable classifications, particularly in relation to higher energy and sugar values.

An additional finding was the significant difference in the percentage of fruits, vegetables and legumes across product subtypes, with the highest median values observed in barista products. Although this component contributes positively to Nutri-Score calculation, its interpretation in plant-based beverages should remain cautious because such values may also reflect formulation choices and the proportion of plant-derived ingredients rather than overall nutritional adequacy. This pattern further supports the notion that formulation-related characteristics may meaningfully shape nutrient profiling outcomes within product subtype categories.

Previous research has shown that Turkish consumers generally perceive plant-based beverages as “healthier” and “lower in calories” than conventional dairy products (23). However, our findings indicate that especially plain/original and barista-type products may exhibit nutritional profiles that contradict this perception. Nutri-Score labeling may help mitigate the so-called “health halo” effect by enabling consumers to recognize the actual nutritional quality of products directly from the front of the package (24, 25). Although Nutri-Score provides a practical summary of the overall nutrient profile of foods and beverages, it should not be interpreted as a complete measure of nutritional adequacy. In plant-based beverages, important attributes such as micronutrient fortification, protein quality, amino acid composition, the origin of sugars, and potentially relevant bioactive compounds are not fully captured by the scoring system. For this reason, Nutri-Score may be useful as a front-of-pack comparative tool, but its interpretation should be accompanied by awareness of these structural limitations.

There is currently no mandatory FOPL system in Türkiye. However, the harmonization process with the European Union and the increasing prevalence of obesity and diabetes make the need for tools that support more informed food choices more evident. From a public health perspective, the present findings highlight that plant-based beverages should not be regarded as nutritionally uniform products. Because these beverages may be perceived by consumers as inherently healthy, simplified front-of-pack information may be useful in helping consumers distinguish between products with more and less favorable nutrient profiles. Although the present study does not directly evaluate consumer behavior or policy implementation, it offers preliminary evidence relevant to discussions on front-of-pack nutrition communication for plant-based beverages in Türkiye.

One of the major strengths of this study is the systematic evaluation of plant-based beverages identified in selected retail outlets in Türkiye using the updated Nutri-Score algorithm. This study represents one of the first investigations in Türkiye to examine plant-based beverages using the 2023 revision of the Nutri-Score system. In addition, the use of real market products, together with the simultaneous evaluation of different botanical sources (such as almond, oat, soy, and coconut) and product subtypes (unsweetened, plain/original, and barista), constitutes a significant contribution to the existing literature. Demonstrating the apparent importance of product subtype in Nutri-Score classification is one of the strengths of this study, as it suggests that not only the raw material but also the formulation itself may play a relevant role in determining nutritional quality.

This study has several limitations. First, all analyses were based on label information and manufacturer-provided data, without laboratory verification. Second, when dietary fiber was not declared and could not be verified, it was recorded as 0 g/100 ml in this label-based analysis. Third, some plant-source subgroups included only a small number of products, limiting interpretability and the robustness of subgroup comparisons. Finally, the sample reflects products identified in selected retail channels during the study period and should not be interpreted as a complete census of the Turkish market.

## 5 Conclusion

In conclusion, among the sampled products, product formulation appeared to be more strongly reflected in Nutri-Score classification than plant source. The findings indicate that plant-based beverages marketed within the same broad category may differ meaningfully in nutritional profile, particularly with respect to energy and sugars. These results should be interpreted cautiously given the limited and uneven subgroup sizes, but they provide preliminary evidence relevant to future discussions on FOPL for plant-based beverages in Türkiye.

## Data Availability

The raw data supporting the conclusions of this article will be made available by the authors, without undue reservation.
